# Foreign Bodies in the Intestines*Read before the Buffalo Medical Club Feb. 15, 1882.

**Published:** 1882-03

**Authors:** Thomas Lothrop


### Clinical Reports.

### Foreign Bodies in the Intestines.*

Reported by Thomas Lothrop, M. D.

Well-authenticated cases of obstruction of the bowels, from the presence of foreign bodies, are sufficiently rare to make the following a case of much professional interest.

Early in December, 1881, I was called to Maggie Joyce, aged 14 years, from whom I gathered the following history: Until within two years the child had enjoyed very good health, and exhibited the sprightliness and vivacity of mind and physical activity usually observed at that age. But from causes which were inexplicable to her parents, she began to fail in strength and to depreciate in weight, thus interfering with her attendance at school. Nothing very satisfactory either to parents or friends was gathered from the physicians who were consulted. During the winter and spring of 1881, the emaciation became more rapid in its progress, the bowels were alternately constipated and open, and every symptom of marasmus was present. In June last, after the administration of a laxative, the patient discharged a cherry stone with the fæces, and continued to expel these stones at intervals of a few days until my first professional visit. At

*Read before the Buffalo Medical Club Feb. 15, 1882.

that time the patient was emaciated to an extreme degree, the pulse was almost imperceptible, the abdomen tympanitic, the evacuations from the bowels occurring four times and often more a day. A stethoscopic examination of the lungs failed to reveal the presence of either inflammatory or tubercular disease of those organs; œdema of extremities was marked, and a passive infiltration of serum in and about the eye-lids gave evidence of great physical prostration and anæmia. Surveying the previous history of the case, with the candid statements made by both patient and mother, I could not doubt that the foreign substances were veritably discharged from the rectum, and that to this important factor should be attributed the causation of the long train of morbid phenomena which had brought the little patient to her present dangerous state. The mother showed me a considerable number of cherry stones, which she stated were only a portion of those gathered from the fæces evacuated by the patient, among which were also a few plum stones, all of which were blackened in their passage through the intestinal canal.

In the extreme condition in which the patient was found at my first visit, but little hope of recovery was held out, and only such remedies prescribed as would support the vital forces already nearly exhausted by the long-continued drain which the frequent alvine evacuations had occasioned. Cod-liver oil supplied the want in nutrition, and bismuth and pepsine assisted in gastric digestion.

The evacuation of cherry and plum stones continued, often daily, sometimes two or three days intervening between the time of their discharge. Sometimes two would be discharged a day; then again, after the lapse of three or four days, three or four stones would come away at a time. The patient failed to respond to the nutrients administered, and gradually sank, death occurring Jan. 30.

Autopsy forty hours after death, performed by Dr. Burt Hoyer, a recent graduate of the Buffalo Medical College, in the presence of Dr. D. Macniel and the writer.

The adipose tissues were almost entirely absorbed; the abdominal cavity was greatly distended with the flatus contained in the intestines, and with serum in the cavity itself. The stomach and small intestines were healthy, until we examined the *ilium* at a point six inches from its junction with the cæcum, where the cause of all the trouble, which brought the patient to her death, was found. The intestine was black, or very dark in its dependent portion, the intestinal walls thickened from inflammatory deposits, and the canal so far contracted that it was almost impervious, etc. At a point about four inches from the cæcum a collection of plum and cherry stones were found, some of which were deeply incased in the intestinal walls. The cæcum at its junction with the ilium, and for an inch below the junction, was thickened, dark and also contained imbedded in its walls the foreign bodies above referred to; the ilio-cæcal valve was nearly obliterated through inflammatory and ulcerative action. Three perforations were also found; one in the cæcum, and two at the ilio-cæcal junction. Within the ileum and cæcum seventeen plum stones, three cherry stones and seven small bones were found. The cæcum and colon were otherwise in a healthy condition, and none of the foreign bodies were found in transitu in the large intestine. The appendix vermiformis was in a healthy condition, and also the portions of the cæcum around its orifice. The internal coats of the ileum and cæcum were ulcerated at points where the foreign bodies were in contact with the mucous surface, while, as stated above, the entire intestine between the distance above named was in such a condition as we would expect from the influences of the inflammatory action, which the foreign bodies must have produced.

The writer cannot vouch for the correctness of the statements of the patient and attendants, although no reasonable doubt can be thrown around the results of the very close and direct inquiries which were instituted in regard to the time at which the plums and cherries were swallowed. With a positiveness, which savored of truthfulness and sincerity, the patient assured me that neither plums nor cherries were eaten during the summer of 1881. The fact that cherry stones were evacuated in June, 1881, before the season of these fruits, corroborates this statement. The circumstances of the family are not such as to warrant the use of canned fruits, now so much used by housekeepers; hence we can safely eliminate this source from which to trace these foreign bodies. The patient again and again affirmed that she had not eaten these fruits since the summer of 1880, or nearly a year prior to the time the stones appeared in the evacuations. There is so much of truthfulness in all other statements made in regard to the history of this case, not only by the sufferer, but by the mother, that I am inclined to accept as truth this assertion. If this be so, then the stones to the number of at least one hundred must have remained in the intestines for nine months before any evidenee of their presence was given. Those that were found in the ileum and cæcum, according to this computation, were retained at least fifteen months after they were swallowed, an instance of tolerance upon the part of the intestinal canal of unusual occurrence and indeed almost incredible.

The case here reported is suggestive of the dangers caused by the presence of foreign bodies in the intestinal canal, introduced through the ingesta, and also of the difficulty of a correct diagnosis unless the foreign substances show themselves in the fæces. The tolerance of the bowels, for a compartively long period, to agencies which cannot be appropriated to the specific purposes of alimentation and nutrition, is a feature forcibly illustrated in this instance. Foreign bodies generally pass through the intestines, with little or no inconvenience and suffering, and unless they are pointed, as in the case of pins or needles, they generally prove innocuous.

The case here reported demonstrates that the quantity of fruit stones (at least one hundred in number) was so great a source of irritation that a few, having become interrupted in their passage along the canal, and probably impacted, produced the intestinal lesions, which finally, after many months of suffering, led to a fatal issue. We could not find any evidence of stricture of the gut, nor is it probable that any such constriction existed prior to the swallowing of the fruit stones, hence it is reasonable to assume that the large number of stones was the chief factor in the problem, rather than the prior existence of any unusual impediment to their passage. The perforations were evidently of very recent date, and may have been the result of post-mortem changes, inasmuch as peritoneal inflammation had not taken place.

In the experience of the practitioner, attention is often directed to the swallowing of foreign substances, and symptoms of their presence in the alimentary canal are rarely if ever noticed. A dose of castor oil or some other laxative will usually in twenty-four or forty-eight hours expel the foreign body and relieve the patient and friends of anxiety. There are exceptions to this rule, and the case I have cited proved to be such, the only fatal result from this cause observed in quite a large experience. Even in this case, unless verified by the autopsy, doubt would have arisen as to the real source of the fruit stones, and to the credibility of the testimony of the patient and her attendants. The differential diagnosis in the various varieties of gastric and intestinal irritation, inflammation and obstruction, must always remain a problem, the solution of which is only made through the autopsical examination.

The license practiced by many in their ingesta, the results of which are daily brought to the attention of the profession, fails to create surprise that the alimentary canal at times yields to the indigestible character of its contents, and puts on a morbid action. How it withstands the abuses of gluttony, the ingestion of indigestible food, the almost limitless varieties of aliments, are certainly greater sources of wonder than is unfolded in the fact that nature fails to appropriate to any purpose in the economy, substances such as the patient, whose case I have reported, originally swallowed.

The case is not without its valuable suggestions, which it would be profitable to heed: First, in dispelling the doubt as to the influence of foreign bodies in the production of diseased action in the intestine, concerning which there exists in the profession a certain amount of skepticism, and, second, as to the dangers which must follow in case the foreign bodies in their passage along the intestinal canal are interrupted in their progress, and thus forms the nidus for fatal disease.

